# Effects of chains squat training with different chain load ratio on the explosive strength of young basketball players’ lower limbs

**DOI:** 10.3389/fphys.2022.979367

**Published:** 2022-08-29

**Authors:** Dongting Jiang, Gang Xu

**Affiliations:** Sports Coaching College, Beijing Sport University, Beijing, China

**Keywords:** power training, training load, basketball youth athletes, chains squat training, explosive strength of lower limbs

## Abstract

The purpose of this study was to explore the effects of the chain squat training (CST) with different chain load ratio (0, 10%, 20% and 30%) on the explosive power of the lower limbs of adolescent male basketball players. Forty-four youth basketball players (age 15.48 ± 0.81 years, body mass 78.86 ± 12.04 kg, height 184.95 ± 6.71 cm) were randomly allocated to one of the four groups: traditional squat training (TST), 10% chains squat training (10% CST), 20% chains squat training (20% CST), and 30% chains squat training (30% CST). Training interventions were performed 2 times per week for 6 weeks, and at the week before (Pre) and after (Post) the 6-week CST program with different chain load ratio, the no-step vertical jump, standing long jump, 15 m shuttle run, 1 R M squat and 30 m sprint test were performed. A 4 (group) × 2 (time) repeated measures analysis of variances (ANOVA) was calculated to show the scatter of each variable, and the Bonferroni’s post-hoc test was used for multiple comparisons, in addition the partial eta-squared (η^2^) was calculated as an estimate of the ES. Significant time × group interaction was noticed for the no-step vertical jump (*p* < 0.001; η^2^ = 0.611), standing long jump (*p* < 0.001; η^2^ = 0.490) and 1 R M squat (*p* < 0.01; η^2^ = 0.333) indicating that better improvements appear in CST compared to TST. However, significant time × group interaction was noted for 15 m shuttle run (*p* < 0.001; η^2^ = 0.428), in favor of TST compared to CST. In addition, the improvements in 30 m sprint were similar between all groups. In conclusion, CST with more chain load has better training effects on lower limb explosive strength and maximum strength, based on the improvement in 1 R M squat and jumping performance. Besides, compared with TST, CST with more chain load might not help to develop better velocity adaptation at higher range of movement.

## Introduction

As a high-intensity sport requiring for both of speed and power, basketball places high demands on athletes in terms of physical abilities, with the explosive ability being the most significant (Ostojic et al., 2006). In adolescence, the young athlete’s body is in a rapid growth and development period, so that it is the golden period to fully tap into the athletes’ physical potential in an efficient and injury-free manner (Faigenbaum et al., 2011). However, in traditional free weight training, the “sticking point” (when the mechanical advantage of the joint angle is at its lowest level) will lead to a partial loss of muscle strength and a slowing of movement speed (Kompf and Arandjelović, 2016). Moreover, the slowing down of movement would also seriously compromise the explosive power gains (Baechle et al., 2008). Therefore, it is suggested that the traditional training methods should be reformed be adjusted to suit the training needs of young athletes (Faigenbaum et al., 2009).

Traditional barbell training is the conventional selection for strength training of young basketball players, which is effective in inducing muscle hypertrophy and strength growth, but not effective in improving lower limb explosive power. However, the long term use of a single means of strength training may lead to the emergence of a plateau in adaptations for athletes (Bompa and Buzzichelli, 2019). Compared to the traditional barbell squat training, the chains squat training (CST) can provide a dual stimulation effect on motor nerves and muscles, and has higher training benefits (Joy et al., 2016). Besides, the CST can help to reduce the loss of movement speed by reducing the weight of load at the moment of “sticking point,” which is also conducive to preventing sports injuries (Ebben and Jensen, 2002). It was widely reported that CST has the advantages in improving the maximum strength, enhancing the power, promoting the sporting performance and sports rehabilitation (Ebben and Jensen, 2002; McCurdy et al., 2009; Walker et al., 2013).

The ratio between variable resistance (VR) and constant resistance (CR) is likely to be a key point affecting training benefits. Paditsaeree et al. (2016) studied the kinematic difference between variable resistance training (VRT) and constant resistance training (CRT), and found that when using the clean pull action, the variable resistance training with 10% VR and 90% CR shows a higher peak power output. Besides, Wallace et al. (2006) pointed out that the VRT with 85% 1 RM (35%VR + 65%CR) would have a higher training benefit for lower limb training. According to experienced trainers, the VRT is the most beneficial to the development of limb maximum strength when VR is set at 15%–20% and CR is set at 70%–90% (Bellar et al., 2011; Andersen et al., 2015; Joy et al., 2016). As the optimum ratio between VR and CR for the power and strength training is mostly derived from the researcher’s own experience, and the training effects of VR with different ratio of VR and CR are still controversial, therefore more comparative experiments are needed to study the young basketball players’ lower limb explosive power training.

With this context, the aim of the present study was conducted to study the effects of 6-week CST with different ratio (0%, 10%, 20%, 30%) between VR and CR on the development of lower limb explosive power in young basketball players. It was hypothesized that the effects of CST with more ratio of VR would be more effective in enhancing young basketball players’ lower limb explosive power.

## Materials and methods

### Participants

Forty four male pubertal basketball players were recruited and given written informed consent for participating in the study. The requirements for all participants were: a) good health status, b) no severe lower extremity injury occurred during the 6 months prior to the study, c) not received systematic lower body strength training during the 3 months prior to the study, e) playing experience ≥3 years (Table 1). The participants were fully informed about the purpose and procedures of the study, and The study protocol was approved by the local Ethics Committee, with all procedures being in accordance with the latest version of the Declaration of Helsinki. To calculate the sample size, a free statistical software (G * Power, v.3.1.9.7, Dusseldorf, Germany) was used. Given the applied a 4 (group) × 2 (time) repeated measures analysis of variance (ANOVA), a medium overall effect size (ES) = 0.3, an alpha-error = 0.05 and the desired power (1-ß error) = 0.8, the total sample size resulted in 36 participants. To reduce the risk of experimental mortality we recruited a larger sample.

**TABLE 1:** Participants characteristics (mean ± SD).

	TST (*n* = 11)	10%CST (*n* = 11)	20%CST (*n* = 11)	30%CST (*n* = 11)	Group comparison (*p* value)
Age (year)	15.55 ± 0.93	15.73 ± 0.79	15.18 ± 0.75	15.45 ± 0.82	0.484
Body mass (kg)	75.82 ± 8.98	81.82 ± 15.14	78.64 ± 9.60	79.18 ± 14.71	0.732
Height (cm)	184.00 ± 4.80	184.27 ± 7.55	184.36 ± 6.58	186.18 ± 8.47	0.885
BMI (kg/m^2^)	22.33 ± 1.82	23.72 ± 3.40	23.12 ± 2.45	22.68 ± 2.83	0.655
Training experience (year)	3.63 ± 0.67	3.73 ± 0.79	3.81 ± 0.60	3.73 ± 0.65	0.941

TST, tradition squat training; 10%CST, 10% chains squat training (10% VR + 90% CR); 20% CST, 20% chains squat training (20% VR + 80% CR); 30% CST, 30% chains squat training (30% VR + 70% CR).

### Design and procedures

As stated above, we examined the influence of four different 6 week squat training interventions on young basketball player’s lower-limb explosive power variables: vertical jumping ability, horizontal jumping ability, change of direction ability, maximum strength, and short-distance sprinting. After the baseline assessment, all participants were randomly allocated to one of the four following groups: traditional squat training (TST, *n* = 11); 10% chains squat training (10%CST, *n* = 11); 20% chains squat training (20%CST, *n* = 11); 30% chains squat training (30%CST, *n* = 11). The load setting of the chain squat training for each participant was calculated according to their group. A 6-week intervention was performed on each group, which consistsed of two sessions per week of TST or CST in 2020, between September and October. The time interval between two training sessions was at least 48 h to ensure adequate recovery of the lower limb muscles and to avoid bad training state or sports injuries caused by fatigue. All participants were tested before and after the 6 week training period to determine the effects of the four training interventions. The baseline assessment was carried out 1 week before the experimental training programs started and the post-training assessment was performed 1 week after the programs finished. The tests performed included a no-step vertical jump, standing long jump, 15 m shuttle run, one repetition maximum (1 RM) squat test, and 30 m sprint. After 3 weeks of training, all participants were again tested with a 1 RM squat and then the load weight of the subsequent 3 weeks of chain squat training was adjusted based on the new 1 RM squat test value.

In order to reduce the risk of training and testing injuries, all training sessions were supervised by an instructor, who acted as the physical and athletic performance specialist to demonstrate and teach the exercise technique involved in this experiment. Besides, during the experimental training intervention, the participants were instructed to refuse other heavy-load lower limb strength training to avoid interference from external factors.

### Training intervention

All participants were first required to completed a warmup consisting of 5 min of sub-maximal running, then did five static stretching movements (spinal twist, supine knee flex, lateral quadriceps extension, semistraddle, step stretch) and five dynamic stretching movements (arm hugs, heel-to-toe walk, lunge walk with eblow to instep, high hurdle step, spiderman crawl). The overall warm-up exercise took about 15–20 min. After the completion of the exercise, the participants entered the formal training with an interval of 2 min.

Firstly, the participants used 50% 1 RM to do squat exercises to achieve the purpose of specific warm-up, 6 times per set, two sets, 2 min interval between sets. As state before, during formal training, the participants were divided into four groups, all of which used 85% 1 RM to do squat exercises, 6 times per set, five sets, 2–3 min between sets. Both groups received the same encouragement to lift with maximal intended concentric velocity and complete the eccentric phase in a controlled manner. After 3 weeks of training, the load intensity of all participants was adjusted according to the change of 1 RM. Each group executed the training load according to the preset training intervention plan, that is, the TST (85% 1 RM, 0% VR + 100% CR), 10% CST (85% 1 RM, 10% VR + 90% CR), 20% CST (85% 1 RM, 20% VR + 80% CR), and 30% CST (85% 1 RM, 30% VR + 70% CR).

After the training, the foam roller was used to relax the lower back, gluteus maximus, gluteus medius, hip rotator, tensor fascia lata, adductor, quadriceps femoris and hamstring muscles. 10 slow rolls were performed at each position for a total of approximately 10 min. Finally, static stretching (step stretch, semistraddle, sitting toe touch, butterfly, wall stretch) was carried out for about 5 min.

### Quantifying chains squat training loads

The load setting of the CST in this experiment was based on the guidelines drawn up by Haff and Triplett (2015). The weight of the VR (chains) was the average of the chains weights at the highest and lowest points of the squat. For example, for the 85% 1 RM (10% VR, 90% CR) used by participants to do CST, first calculated the weight of 85% 1 RM for a squat without chains, assuming that the weight of 85% 1 RM was 100 kg, thus the weight of VR (chains) was 10 kg ( $\frac{VR(Highest\;point)+VR(Lowest\;point)}{2}=10kg$ ), and the weight of CR (barbell) was 90 kg.

After grouping, the participants for CST (10% CST, 20% CST, 30% CST) were measured for the weight of the chains. In the measurement, the force platform (Kistler 9281CA, Switzerland, 1,000 Hz) was placed under the chains at both ends of the barbell bar, and the length of the chains was adjusted according to the height of participant to ensure that part of the chains could fall on the ground. First the participant carried the barbell (without weight plate) in a standing position, paused for 2 s, recorded the weight of chains (highest point of VR); then the participant performed squat to the lowest point of motion, paused for 2 s, recorded the weight of chains (lowest point of VR), and calculated the weight of VR. Repeated the measurement operation three times, took the average of the three VR values, and adjusted the weight of chains according to the preset weight of VR. The instructor recorded the size and number of chains at the preset weight of VR and made a record of corresponding chains configuration for each participant.

### Measurements

One week before the baseline assessment, the instructors provided technical guidance to all participants to help them become familiar with the test procedures. The baseline assessment was carried out 1 week before the experimental training programs started and the post-training assessment was performed 1 week after the programs finished. The tests were conducted in the following order: no-step vertical jump, standing long jump, 15 m shuttle run, 1 RM squat, 30 m sprint. Before testing, all participants should complete the warmup, which consisted of 10 min of sub-maximal running and dynamic stretching, with the rest intervals between tests of 10min.

### No-step vertical jump

The tests were performed using a height measurement system (Vertec; Hoggan, Los Angeles, United States) to quantify the vertical jumping ability of participants. In the test, the participants started from a standing position and were instructed to perform a downward movement followed by complete extension of the lower limbs and the amplitude of the countermovement was freely determined to avoid changes in the jumping coordination pattern, and tapped the accessible blade with the fingertip at the highest point. The participants were required to complete concentric contraction as soon as possible after the eccentric phase, which was beneficial to the completion of a rapid stretch shortening cycle action.

Participants were also required to land on both feet at the same time. Similarly, each participant completed three trials with 30s of rest between trials, and also the best result of the three trials of the absolute height reached was recorded for further analysis.

### Standing long jump

Before this test, the participants stood behind the baseline. After hearing the instructions of the tester, the participants swung arms backwards and squatted, then swung arms forward and upward quickly and tried to jump forward. After completing the jump, the results for the athlete landed on both feet are considered to be valid, otherwise it must be measured again. The tester measured the distance from the baseline to the heel (the foot closer to the baseline) with a tape measure (Deli-8218; Deli, Ningbo, China). Each participant completed three trials with 30 s of rest between trials, and the best result of the three trials was recorded for further analysis.

### Fifteen metre shuttle run

Before the tester whistled for starting, all participants stood behind the baseline, and their toes were not allowed to cross the baseline. After hearing the whistle, the participants sprinted forward 15 m at maximum speed to the turn-back line, and upon arrival, quickly turned and accelerated back to the baseline. One foot of the participant, either the left or right one, must cross the baseline or the turn-back line before changing of direction. The participants were required to complete a 15 m shuttle run for three times, and the timing was stopped when their feet crossed the baseline at the last return. A false start or no touching the line with foot would be considered an invalid result. Photocells (Smart Speed, Fusion Equipment, Brisbane, AUS) were positioned at the baseline to measure the time taken to complete the course. All participants performed three times, each separated by 2 min of rest, and the best result was used for further analyses.

### One repetition maximum squat

The test procedure of 1 RM squat was carried out according to the protocol provided by the National Strength and Conditioning Association (Haff and Triplett, 2015). First, specific squat warm-up repetitions (5–10 times) were performed by participants at a load of 20%, 40%, and 60% of their estimated 1 RM. The first testing load was set to be 80% of the estimated 1 RM and was increased by 5–10 kg for each subsequent trial. This process was repeated until failure. The rest interval between two successful sets was 5 min. The maximum strength was determined within five attempts to avoid fatigue and accuracy of test results.

### Thirty metre sprint

Two pairs of photocells (Smart Speed, Fusion Equipment, Brisbane, AUS) were positioned at the starting line and at the distance 30 m along the sprinting course respectively. The participants initiated the sprint from a standardized starting position which was 0.5 m behind the starting line. All participants performed three maximal 30 m sprints, each separated by 2min of rest. Three trials were conducted and the best result was used for further analyses.

### Data analysis and statistics

All values were presented as mean ± standard deviation (SD). The homogeneity of variance across groups was verified using the Levene’s test, whereas the normality of distribution of the data was examined with the Kolmogorov-Smirnov test. A 4 (group) × 2 (time) repeated measurement analysis of variances (ANOVA) was calculated for each variable, and the Bonferroni’s post-hoc test was used for multiple comparisons. Further, the partial eta-squared was calculated as an estimate of the ES, with the values of 0.01, 0.06, and above 0.14 considered as small, medium, and large, respectively. Percentage changes were calculated as [(post training value–pretraining value)/pretraining value] × 100. Absolute and relative reliability was assessed using the coefficient of variation (CV) and a two-way random intraclass correlation coefficient (ICC), respectively. Percentage change was calculated for all variables and subsequently compared to CV values to determine whether changes in explosive strength performance was greater than the test variance, thus providing an indication of whether true change occurred for each participant (Bishop et al., 2021). The level of significance was set at *p* < 0.05, and statistical analysis was conducted using SPSS 26.0 (IBM, United States) and Office Excel 2010 (Microsoft Corporation, United States).

## Results

The statistical testing confirmed the normal distribution of the data set in each group and the assumption of homogeneity of variance between the groups for all variables. The analyses of variance (ANOVAs) revealed that there were no statistically significant differences between the training groups in any of the lower limb explosive power variables tested at baseline (*p* > 0.05). The results are outlined in Table 2.

**TABLE 2:** Descriptive statistics (mean ± SD), percentage changes (∆%), and effect size (ES) in five lower limb explosive power variables pre and post-intervention between groups.

Variables	Group	Pre	Post	CV(%)	ICC	Δ(%)	η^2^	Main and interaction effects
No-step vertical jump (cm)	TST	291.45 ± 3.85	292.64 ± 4.16	0.55	0.784	0.41 ± 0.46	0.089	Interaction: *p* < 0.001; η^2^ = 0.611
	10% CST	291.09 ± 5.33	293.95 ± 5.29*	0.62	0.855	0.99 ± 0.60	0.366	Group: *p* = 0.541; η^2^ = 0.052
	20% CST	291.23 ± 5.05	296.36 ± 5.11*	0.43	0.931	1.77 ± 0.88	0.650	Time: *p* < 0.001; η^2^ = 0.829
	30% CST	291.32 ± 6.92	298.77 ± 6.31*	0.42	0.969	2.57 ± 0.75	0.796	
Standing Long Jump (cm)	TST	247.05 ± 1.78	249.23 ± 2.55*	0.51	0.755	0.88 ± 0.71	0.255	Interaction: *p* < 0.001; η^2^ = 0.490
	10% CST	247.22 ± 4.49	250.09 ± 4.01*	0.60	0.831	1.17 ± 0.98	0.371	Group: *p* = 0.614; η^2^ = 0.044
	20% CST	246.77 ± 3.34	252.14 ± 3.37*	0.48	0.895	2.18 ± 0.89	0.674	Time: *p* < 0.001; η^2^ = 0.840
	30% CST	246.50 ± 3.44	253.18 ± 3.61*	0.52	0.905	2.71 ± 0.57	0.763	
15 m Shuttle Run (s)	TST	19.56 ± 0.28	18.69 ± 0.40*^,§^	1.66	0.721	4.46 ± 1.56	0.775	Interaction: *p* < 0.001; η^2^ = 0.428
	10% CST	19.49 ± 0.29	18.86 ± 0.40*	1.83	0.687	3.21 ± 1.27	0.640	Group: *p* = 0.516; η^2^ = 0.055
	20% CST	19.52 ± 0.45	18.96 ± 0.50*	1.82	0.688	2.89 ± 1.21	0.591	Time: *p* < 0.001; η^2^ = 0.864
	30% CST	19.51 ± 0.45	19.21 ± 0.34*	1.84	0.615	1.53 ± 0.91	0.292	
1 RM Squat (kg)	TST	92.27 ± 4.10	93.18 ± 5.13	2.61	0.896	1.04 ± 4.69	0.018	Interaction: *p* < 0.01; η^2^ = 0.333
	10% CST	90.91 ± 4.91	94.55 ± 4.72*	2.75	0.912	4.12 ± 4.59	0.230	Group: *p* = 0.364; η^2^ = 0.760
	20% CST	91.36 ± 3.23	96.82 ± 5.13*	2.85	0.900	5.93 ± 2.81	0.402	Time: *p* < 0.001; η^2^ = 0.628
	30% CST	91.82 ± 4.05	99.09 ± 4.37*^,#^	2.80	0.930	7.96 ± 2.94	0.545	
30 m Sprint (s)	TST	4.55 ± 0.15	4.43 ± 0.16*	1.58	0.713	2.72 ± 3.58	0.173	Interaction: *p* = 1.000; η^2^ = 0.000
	10% CST	4.57 ± 0.15	4.44 ± 0.17*	1.47	0.761	2.79 ± 3.78	0.181	Group: *p* = 0.951; η^2^ = 0.008
	20% CST	4.59 ± 0.22	4.46 ± 0.20*	1.44	0.837	2.75 ± 2.57	0.177	Time: *p* < 0.001; η^2^ = 0.480
	30% CST	4.58 ± 0.23	4.46 ± 0.17*	1.56	0.824	2.68 ± 2.32	0.173	

*p < 0.05, significantly different from Pre.

^§^ *p* < 0.05, significantly different from 30% CST (*p* = 0.035).

^#^ *p* < 0.05, significantly different from TST (*p* = 0.040).

### No-step vertical jump

After 6 weeks of training intervention, significant improvements were found in the 10%CST (2.86 cm; 0.99%; *p* < 0.001; η^2^ = 0.366), 20%CST (5.13 cm; 1.77%; *p* < 0.001; η^2^ = 0.650) and 30% CST (7.45 cm; 2.57%; *p* < 0.001; η^2^ = 0.796) in the no-step vertical jump test. However, the post hoc analyses showed no between-group differences in no-step vertical jump height at post-intervention as in the results of standing long jump.

### Standing long jump

After 6 weeks of training intervention, all the groups i.e., TST, 10%CST, 20%CST and 30%CST showed a significant improvement in the standing long jump test (2.18 cm, 0.88%, *p* = 0.001, η^2^ = 0.255; 2.87 cm, 1.17%, *p* < 0.001, η^2^ = 0.371; 5.37 cm, 2.18%, *p* < 0.001, η^2^ = 0.674 and 6.68 cm, 2.71%, *p* < 0.001, η^2^ = 0.763, respectively). However, the post hoc analyses showed no between-group differences in standing long jump distance at post-intervention.

### Fifteen metre shuttle run

After 6 weeks of training intervention, all the groups i.e., TST, 10% CST, 20% CST and 30% CST showed a significant improvement in the 15 m shuttle run test (0.87s, 4.46%, *p* < 0.001, η^2^ = 0.775; 0.63 s, 3.21%, *p* < 0.001, η^2^ = 0.640; 0.56 s, 2.89%, *p* < 0.001, η^2^ = 0.591 and 0.30 s, 1.53%, *p* < 0.001, η^2^ = 0.292, respectively). Besides, the post hoc analyses revealed between-group differences (*p* = 0.035) in times for the 15 m shuttle run test. In addition, from the results, it is found thatthe 15 m shuttle run was significantly faster for the TST (0.87 s, 4.46%, *p* < 0.001, η^2^ = 0.775) than for the 30%CST (0.30 s, 1.53%, *p* < 0.001, η^2^ = 0.292) at post-intervention.

### One repetition maximum squat

After 6 weeks of training intervention, significant improvements were found in the 10% CST (3.64 kg; 4.12%; *p* = 0.01; η^2^ = 0.230), 20% CST (5.46 kg; 5.93%; *p* < 0.001; η^2^ = 0.402) and 30% CST (7.27 kg; 7.96%; *p* < 0.001; η^2^ = 0.545) in the 1 RM squat test. Besides, the post hoc analyses revealed between-group differences (*p* = 0.040) existed in weights for the 1 RM squat test. Improvement in 1 RM squat was significantly higher for the 30% CST (7.27 kg; 7.96%; *p* < 0.001; η^2^ = 0.545) than that for the TST (0.91 kg; 1.04%; *p* = 0.392; η^2^ = 0.018) at post-intervention.

### Thirty metre sprint

After 6 weeks of training intervention, all the groups i.e., TST, 10% CST, 20% CST and 30% CST showed a significant improvement in the 30 m sprint test (0.12 s, 2.72%, *p* = 0.006, η^2^ = 0.173; 0.13 s, 2.79%, *p* = 0.005, η^2^ = 0.181; 0.13 s, 2.75%, *p* = 0.006, η^2^ = 0.177 and 0.12 s, 2.68%, *p* = 0.006, η^2^ = 0.173, respectively). However, the post hoc analyses showed no between-group differences in 30 m sprint time at post-intervention.

**FIGURE 1:**
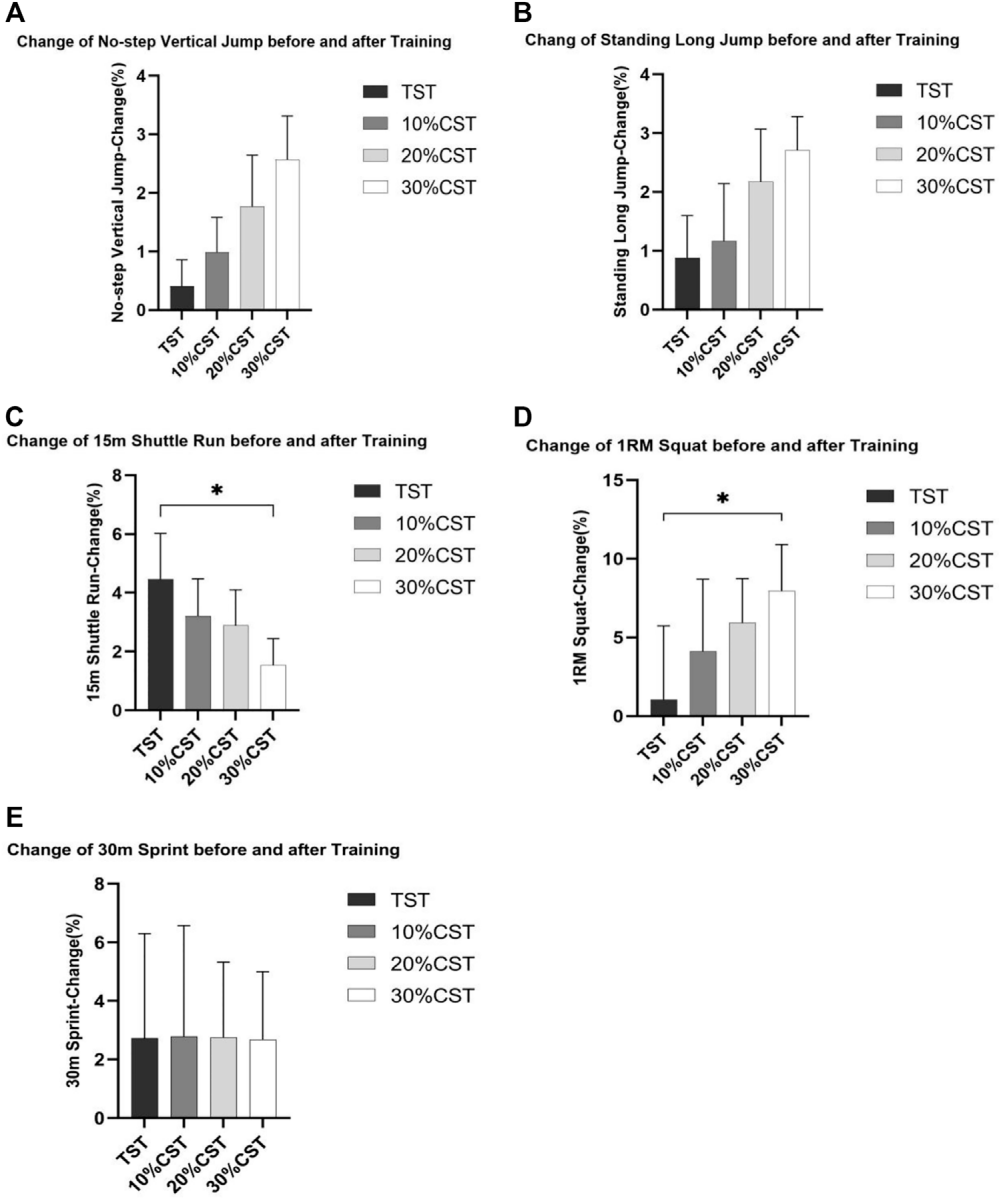
Percentage changes (Δ%) of five lower limb explosive power variables with the increase of the weight of VR (chains): No-step Vertical Jump **(A)**, Standing Long Jump **(B)**, 15 m Shuttle Run **(C)**, 1 RM Squat **(D)**, 30 m Sprint **(E)**. *p < 0.05, significant differences between groups.

Figure 1 indicated that with the increase of the weight of VR (chains), the percentage changes of 1 RM squat, standing long jump and no-step vertical jump showed an increasing trend, and the changes of 15 m shuttle run showed a decreasing trend, while the changes of 30 m sprint had no obvious trend.

## Discussion

As the basis of explosive power, the maximum strength plays a vital role in the transformation and development of special strength in the specific preparation period (Bompa and Buzzichelli, 2019). After 6 weeks of training intervention, with the increase of the weight of VR, the percentage change of 1 RM squat showed an increasing trend. The adaptation and improvement of strength training mainly come from muscular adaptation (muscle hypertrophy) and neurogenic adaptation, and neurogenic adaptation occurs before muscular adaptation (Sale, 2008). Muscle hypertrophy requires a certain concentration of testosterone, however, since the participants are all 14–17 years old, the testosterone concentration in their bodies at this age is limited (Fukunaga et al., 1992), therefore, the improvement of the maximum strength is mainly due to the improvement of neurogenic adaptation (motor unit recruitment, motor unit synchronization and firing rate of motor nerves are improved). The main physiological mechanism of CST is to achieve the effect of stimulating motor nerves through the change of load weight (Joy et al., 2016). Therefore, a higher proportion of VR would lead to a greater change in load weight in CST and a better stimulation effect on motor nerves. Nervous adaptation is the basis of sports performance, and increasing neural drive is very important for exerting muscle strength and explosive force (Aagaard et al., 2002). At the same time, a higher proportion of VR also means that the instability of training becomes greater, which can induce greater involvement of relevant stabilizing muscles and create favourable conditions for the active muscles to exert force, thus facilitating the improvement of intermuscular coordination (Berning et al., 2008). Anderson and Behm (2004) also found that although there are no significant differences in overall EMG activity between the stable and unstable protocols, resistance training on an unstable surface may still force limb musculature to play a greater role in joint stability.

Basketball players’ lower limb jumping ability is very important for completing various offensive and defensive techniques (e.g., rebound, defensive slide, running jump shot, and jump shot). Therefore, jumping tests are widely used in the physical fitness test of basketball, and standing long jump and no-step vertical jump are tests of athletes’ horizontal and vertical jumping abilities respectively, which emphasizes the evaluation of the stretch-shortening cycle (SSC) for athletes’ lower limb muscles. After 6 weeks of training intervention, with the increase of the weight of VR, the percentage changes of standing long jump and no step vertical jump also showed an increasing trend. The power training emphasizes the sufficient combination of strength and speed. Due to the “sticking point,” the TST would lead to loss of part of the movement speed. Ebben and Jensen (2002) stated that there are no differences in integrated electromyography (I-EMG) and ground reaction force (GRF) during the eccentric or concentric phase for TST and 10%CST. The results of this study showed that 20% CST and 30% CST were relatively more effective in improving jumping ability. The 20% CST and 30% CST seem to be able to minimize the loss of movement speed while maintaining the load weight, providing progressive resistance to the muscles as the joint angle changes (when the mechanical advantage of the joint angle gradually improves), which would help the muscles produce a higher level of muscle strength during the concentric contraction and improve the rate of force development (RFD) (Findley et al., 2004; Berning et al., 2004). Another important factor improving RFD is the shortening of SSC (Rhea et al., 2009). The SSC consists of three phases: the eccentric contraction phase, the amortization phase and the concentric contraction phase, of which the amortization phase is the key link affecting the power output and should be as short as possible (Cavagna, 1977). With the increase of the weight of VR, the load weight at the lowest point of squat will decrease, meaning that the time of muscle eccentric contraction to the lowest point will be shorter and the muscle is stretched faster, which is beneficial to the occurrence of stretch reflex (Nicol and Komi, 1998). Besides, Soria-Gila (2015) also found that the acceleration of movement speed at the lowest point of the CST improves the SSC effect and enhances the muscle strength output at the beginning of the concentric contraction.

The transition between attack and defence in basketball matches is very rapid and frequent. The fast-paced characteristic of the game requires athletes to have excellent ability of acceleration, deceleration and change of direction (COD) on the basketball court (length: 28 m; width: 15 m). Hence, the 15 m shuttle run is a convenient specific performance test to measure above abilities, and the 30 m sprint is an evaluation of basketball players’ acceleration ability. After 6 weeks of training intervention, with the increase of the weight of VR, the percentage changes of 15 m shuttle run showed a decreasing trend, and the changes of 30 m sprint had no obvious trend. In theory, CST could result in better performance in the COD phase through the improvment in SSC. Because that as the bar is descended, more chains collect to the floor, reducing the load (McCurdy et al., 2009), which allow the participant to perform higher speed eccentric phase more easily. The effect of SSC is more obvious in fast stretch than in slow stretch (Nicol and Komi, 1998). However, load reduces during descending means that the external load on the muscle decrease in the eccentric contraction phase, which is different from the variation of the external load during deceleration phase in the 15 m shuttle run test. Häkkinen et al. (1985) reported increased electromyographic activity and a controlled increase in velocity during eccentric actions. In another study, Cronin et al. (2003) concluded that VRT using elastic bands attached to a jump squat machine induced greater electromyographic activity in eccentric contractions compared with traditional training methods. Hence, the physiological characteristics of the eccentric contraction phase is different between VRT and TST, the participant of TST may be more adaptable to do eccentric contraction to slow down with constant external load, which is more similar to the controlment of inertia (CR) during deceleration phase. On the other hand, as TST is limited by the sticking region, the movement velocity would increase greatly once the participants overcome that region, and participant would also received the encouragement to lift with maximal intended concentric velocity during the training intervention, therefore participants in TST likely would have adapted to faster movement speed at higher range of movement, which is the joint angles of the lower limb during COD and sprints. In contrast, CST would likely be slowing down or maintain same lifting velocity as they get past the sticking region, hence, they might have adapted to slower contraction velocity at the upper range of movement. Stevenson et al. (2010) stated that both peak and mean velocities in the concentric phase were significantly greater in the CRT than in the VRT, although VRT could increase the overall movement speed, it mainly improved the speed of the eccentric phase, which may limit the development of the speed of the concentric phase. Therefore, based on above statements, we can infer that CST might not provide the participant with better velocity adaptation at higher range of movement, which may magnify the restriction of concentric contraction speed in the sticking region. While CST might have more advantages at lower range of movement by means of SSC. Moreover, both no-step vertical jump test and standing long jump test are allowed to use preferred range of joint angle to avoid velocity loss, so the participants in CST performed better. Finally, COD and sprinting are complex abilities that are affected by more than strength and power alone. Hence, improved strength and power may not lead to significant improvement in these activities.

## Limitations of the study

The main limitation of our study is the lack of EMG index to show in detail the work of relevant muscles. This would help to explore the training effects and training mechanism of CST with different chain load. Another limitation is the lack of long-term effects of CST, which limits the in-depth comparative analysis. Although CST with more chain load showed better training effect in short term, it was not conducive to the improvement of muscle eccentric contraction ability, therefore it is necessary to further explore its long-term training effect. Another limitation may be that little research is available on the training effects of CST with different chain load, and the related researches of the range of chain load are still seldom. Finally, because that CST with more ratio of VR (e.g., 30% CST ) would lead to greater instability and difficulty, although four groups used the same 85% 1 RM to do exercise, the 30% CST group might challenge a higher intensity.

## Conclusion

In the short-term training intervention, CST with more chain load has relatively better training effects on lower limb explosive strength and maximum strength, based on the improvement of neural adaptability. Therefore, it is suggested that 20% CST or 30% CST should be used in the specific preparatory phase and pre-competition phase to promote the transformation of general strength to special strength and also the improvement of lower limb explosive strength. CST might have more advantages at lower range of movement by means of SSC. While, compared with TST, CST with more chain load might not help to develop better velocity adaptation at higher range of movement. Youth basketball physical fitness training emphasizes gradual and comprehensive development. Therefore, according to the different training phases, the load configuration of CST should be flexibly adjusted to achieve different training objectives and help athletes obtain the best training effect.

## Data Availability

The original contributions presented in the study are included in the article/Supplementary Material, further inquiries can be directed to the corresponding author.
